# INO80 regulates promoter-associated R-loops to coordinate transcription and maintain genome stability in embryonic stem cells

**DOI:** 10.1186/s40659-025-00666-7

**Published:** 2026-01-03

**Authors:** Hoseong Lim, Ohbeom Kwon, Hyeonwoo La, Hyeonji Lee, Heeji Lee, Jeong-Tae Do, Hyuk Song, Youngsok Choi, Kwonho Hong

**Affiliations:** Department of Stem Cell and Regenerative Biotechnology and Institute of Advanced Regenerative Science, Konkuk University, Seoul, 05029 Korea

**Keywords:** INO80, Embryonic stem cells, R-loop homeostasis, Chromatin remodeling, Transcription-replication conflict, Genome stability, Hydroxyurea, Apoptosis, Cell cycle regulation

## Abstract

**Background:**

Embryonic stem cells (ESCs) possess a distinctive cell cycle structure and chromatin landscape that necessitate precise coordination between transcription and DNA replication. The chromatin remodeler INO80 regulates both transcription and genome stability; however, its role in R-loop homeostasis in ESCs remains incompletely understood. R-loops, which are formed by RNA:DNA hybrids, play regulatory roles but can also induce replication stress and genomic instability if unregulated.

**Results:**

We investigated the effects of INO80 loss on R-loop dynamics, replication stress responses, and transcriptional regulation in mouse ESCs. INO80-deficient cells exhibited a global reduction in steady-state R-loops under normal conditions, with the most pronounced loss at promoters enriched for pluripotency-associated transcription factors. R-loop levels were restored under hydroxyurea-induced replication stress, indicating that INO80 is dispensable for stress-induced R-loop accumulation but is required for maintaining physiological R-loops. Cleavage Under Targets and Tagmentation and chromatin immunoprecipitation sequencing revealed that several R-loop regions overlap with INO80-binding sites and are linked to transcriptional activation and genome maintenance. Transcriptome analysis showed that INO80-stabilized R-loops are associated with genes involved in cell cycle progression, DNA repair, and chromatin organization, whereas INO80 loss results in altered transcriptional programs, G1 arrest, and increased apoptosis. These findings indicate that INO80 stabilizes functional R-loops that support transcriptional regulation and genomic integrity.

**Conclusions:**

Our study identifies a previously unrecognized role for INO80 in preserving functional R-loops in ESCs. By stabilizing promoter-associated R-loops and coordinating transcription during cell cycle progression, INO80 maintains both transcriptional regulation and genome stability. These findings underscore the importance of chromatin remodeling in maintaining R-loop homeostasis and suggest that INO80 dysfunction may have implications for stem cell identity and genomic integrity.

**Supplementary Information:**

The online version contains supplementary material available at 10.1186/s40659-025-00666-7.

## Background

Embryonic stem cells (ESCs) exhibit a cell cycle profile that differs markedly from that of somatic cells and is characterized by a notably abbreviated G1 phase and an extended S phase [1]. These features are closely linked to the maintenance of pluripotency and rapid proliferation [2, 3]. ESCs sustain high cyclin-dependent kinase (CDK) activity and minimal levels of CDK inhibitors (CKIs), thereby enabling a rapid transition into DNA replication [4–6]. Chromatin architecture and its regulatory components are increasingly recognized as pivotal determinants of ESC cell cycle progression and transcriptional control [7].

ATP-dependent chromatin remodeling complexes, such as INO80, play essential roles in modulating chromatin structure to regulate transcription, DNA replication, and DNA repair [8–30] In particular, the INO80 complex has been implicated in pluripotency maintenance, early development, and cellular stress responses [31] INO80 is enriched at transcription start sites (TSSs) and modulates gene expression in a context-dependent manner, acting either to promote or suppress transcription depending on chromatin architecture and associated transcriptional regulators [31]. Previous studies have shown that INO80 deletion in ESCs leads to impaired proliferation, defective differentiation, and embryonic lethality [32].

Previous work from our group and others has demonstrated that INO80 is essential for ESC proliferation, cell cycle progression, and survival, in part through transcriptional repression of G1 checkpoint regulators such as *Cdkn1a* and *Rb1* [21, 32, 33]. Loss of INO80 leads to prolonged G1 phase and increased apoptosis in ESCs, particularly under primed ESC conditions. Despite these findings, the full spectrum of INO80’s functions in preserving genomic integrity during active proliferation remains incompletely understood.

A key source of genomic instability in proliferating cells is transcription-replication conflicts (TRCs), which can lead to stalled replication forks and DNA damage [34, 35]. A major structural contributor to these conflicts is the R-loop, a three-stranded structure composed of an RNA:DNA hybrid and displaced single-stranded DNA (ssDNA) [36]. While R-loops play regulatory roles in transcription and chromatin organization, their accumulation can induce replication stress and DNA breaks [37, 38].

Recent evidence suggests that conflicts between transcription and replication machineries contribute significantly to genomic instability, particularly through the formation of R-loops, which, while sometimes functional [39–42], can impede replication fork progression [43]. Accumulation of unresolved R-loops is a potent source of replication stress and DNA damage [44]. Chromatin remodelers have emerged as key modulators of R-loop dynamics. In yeast and mammalian cells, the INO80 complex has been shown to safeguard replication fork stability and promote fork recovery under stress conditions, in part by resolving R-loops [20, 30, 45]. However, in the context of ESCs, we observed an unexpected reduction in R-loop levels following INO80 deletion, in contrast to previous findings in somatic and cancer cell models. This outcome led us to hypothesize that INO80’s role in R-loop regulation may be highly context-dependent, shaped by the distinct chromatin landscape and transcriptional dynamics characteristic of ESCs. To address this, we investigated how INO80 loss affects R-loop dynamics, replication stress responses, and cell cycle progression in ESCs.

## Methods

### Cell culture

Control and *Ino80* knockout (*Ino80* iKO) ESCs and MEFs were established in our laboratory [32]. ESCs were maintained under serum-free 2i/LIF culture conditions (2i). MEFs were cultured in high-glucose Dulbecco’s modified Eagle’s medium (DMEM) supplemented with 10% fetal bovine serum (Welgene) and penicillin–streptomycin (Gibco) at 37 ℃ in a humidified incubator with 5% CO_2_.

### RNA:DNA hybrid quantification

Genomic DNA was extracted using the Genomic DNA Kit (Qiagen, Hilden, Germany) according to the manufacturer’s instructions. Approximately 5 μg of purified DNA was divided into two tubes. One was treated with RNase H (10 U per μg DNA; M0297L, NEB, Ipswich, MA, USA), whereas the other was remained untreated as a control. Then, the RNase H-treated and -untreated samples were serially diluted and loaded onto a positively charged nylon membrane (Hybond N+, RPN303B; Cytiva, Marlborough, MA, USA) at 500, 300, and 100 ng per dot, using a MiniFold-1 dot blot apparatus (Cytiva). The DNA was then crosslinked to the membrane by baking at 80 ℃. The membrane was blocked with 5% (w/v) skim milk in Tris-buffered saline with 0.05% Tween-20 for 1 h at room temperature, followed by incubation with primary antibodies specific to double-stranded DNA (dsDNA; Abcam; ab27156) or RNA:DNA hybrids (S9.6; Sigma; MABE1095). The membrane was subsequently incubated with a horseradish peroxidase-conjugated secondary antibody (goat anti-mouse IgG, 1:5000 dilution; Santa Cruz). Chemiluminescent signals were detected using the WEST-Queen Western Blot Detection System containing an enhanced chemiluminescence substrate (Bio-Rad) or the SuperSignal™ West Femto Maximum Sensitivity Substrate (Thermo Scientific) and visualized via autoradiography.

### Analyses of cell proliferation, cell cycle, and apoptosis

Cell numbers were determined using an automated cell counter (EVE™; NanoEntek, Seoul, Republic of Korea). For cell cycle analysis, cells were fixed in 80% ethanol at 4 ℃ for approximately 16 h. After fixation, cells were washed with ice-cold Dulbecco’s phosphate-buffered saline (DPBS) and treated with RNase A (R6148; Sigma-Aldrich, St. Louis, MO, USA) for 2 h. Subsequently, cells were stained with propidium iodide (PI; 556,463; BD Pharmingen, Franklin Lakes, NJ, USA) and analyzed using flow cytometry on a CytoFLEX system (Beckman Coulter, Brea, CA, USA). To assess apoptotic cell populations, cells were stained with an FITC-conjugated Annexin V Apoptosis Detection Kit I (556,547; BD Pharmingen) according to the manufacturer’s instructions, followed by flow cytometric analysis.

### Immunofluorescence

Cells were fixed with 4% paraformaldehyde in PBS for 5 min at room temperature (RT), followed by permeabilization in 0.1% Triton X-100 in PBS for 5 min at RT. After permeabilization, cells were blocked for 1 h at RT in PBS containing 3% donkey serum and 2% bovine serum albumin. Primary antibody incubation was performed overnight at 4 °C in the blocking solution. The following primary antibodies were used: S9.6 (MABE1095; Merck Millipore, Darmstadt, Germany), γ-H2AX (#NB100-384; Novus Biologicals, Colorado, USA), and Oct-3/4 (sc-8628; Santa Cruz Biotechnology, Texas, USA). After three washes with 0.1% Tween-20 in PBS, cells were incubated with appropriate secondary antibodies for 1 h at RT. Nuclear staining was performed using DAPI diluted 1:1000 in PBS. Fluorescent images were acquired using a Zeiss LSM800 confocal microscope.

### R-loop CUT&Tag and downstream data analyses

R-loop profiles were captured using the R-loop Cleavage Under Targets and Tagmentation (CUT&Tag) R-loop Assay Kit (Active Motif, 53,167) according to the manufacturer’s instructions. Briefly, 2 × 10^5^ cells were harvested and washed twice with ice-cold Dig-wash buffer supplemented with protease inhibitors. Washed cells were mixed with Concanavalin A beads in binding buffer and incubated at room temperature for 10 min on a nutator. Bead-bound cells were collected using a magnetic stand and resuspended in complete antibody buffer. RNA:DNA hybrid antibody (clone S9.6, 2 μg) was added, and the mixture was incubated overnight at 4 ℃ on a nutator. Following primary antibody binding, the cells were separated using a magnetic stand and washed three times with ice-cold Dig-wash buffer. The pellets were then resuspended in Dig-wash buffer containing secondary antibody (1:100 dilution) and incubated for 2 h at room temperature on a nutator. After incubation, cells were washed three times with Dig-wash buffer using a magnetic stand. For tagmentation, pA-Tn5 transposomes were diluted 1:100 in complete Dig-300 buffer and added to the cells. The mixture was incubated at 37 ℃ for 1 h. To stop tagmentation and solubilize DNA fragments, 0.5 M EDTA, 10% SDS, and proteinase K (10 μg/μL) were added, and the samples were incubated at 55 ℃ for 1 h in a thermal cycler with the heated lid set to 65 ℃. DNA was then eluted, and libraries were amplified using Illumina i5/i7 indexed primers, followed by SPRI bead clean-up.

CUT&Tag datasets were processed as follows: sequencing reads were trimmed using fastp (v0.23.4) [46] and aligned to the mouse genome (mm10) using Bowtie2 (v2.5.4) [47]. Raw BAM files were filtered using SAMtools (v1.19.2) [48] to remove low-quality and unmapped reads (flag 0 × 4), retaining only properly paired reads (flag 0 × 2) with a MAPQ ≥ 10. To minimize PCR duplicates, BAM files were processed with samtools fixmate, coordinate-sorted, and deduplicated using samtools markdup − r. To eliminate mitochondrial DNA, reads aligning to chromosome chrM were removed using samtools view. Reads mapping to ENCODE mm10 blacklist regions were removed using bedtools intersect (v2.31.1) [49]. Coverage tracks were generated using deepTools (v3.5.5) bamCoverage [50]. Peak calling was performed using MACS3 (v3.0.2) callpeak [51].

### Public data analysis

Both RNA sequencing (RNA-seq) and chromatin immunoprecipitation sequencing (ChIP-seq) datasets were obtained from the same GEO superfamily (GSE158545) in the NCBI Gene Expression Omnibus [27]. RNA-seq data were derived from GSE158544, which includes INO80 wild-type and knockout ESC samples cultured in 2i medium, and ChIP-seq data were obtained from GSE158533, containing INO80 chromatin occupancy profiles generated under the same 2i condition. RNA-seq reads were trimmed using fastp and aligned to the mouse genome (mm10) with the STAR aligner. Read counts per gene were quantified using featureCounts (v2.1.1) [52]. Genes with low expression levels were filtered by calculating Counts per Million (CPM), retaining genes with CPM ≥ 1 in at least one sample. The trimmed mean of M-value normalization was applied to correct for library size differences across samples. After filtering and normalization, the resulting set of expressed genes was used as the background gene set for subsequent enrichment analyses. The INO80 ChIP-seq datasets were processed in parallel using the same computational pipeline described above for CUT&Tag, including read trimming, alignment, filtering, deduplication, and peak calling with MACS3. Normalized signal tracks were also generated using deepTools for downstream integration and comparative analysis with the R-loop CUT&Tag profiles.

### Functional enrichment analysis

Genomic regions were annotated using ChIPseeker (v1.44.0) [53] with the mm10 mouse genome annotation. Gene Ontology (GO) enrichment analysis was performed using DAVID [54, 55].

### Transcription factor motif enrichment analysis

Motif enrichment analysis was performed using Hypergeometric Optimization of Motif EnRichment (HOMER; v5.1) [56]. R-loop-rich regions were used as the input set, and corresponding non-R-loop segments served as the background. P-values, q-values, and fold enrichment were computed using HOMER with hypergeometric and binomial models.

### Statistical analysis

Statistical analyses were conducted using GraphPad Prism software (v8.0.2; GraphPad Inc., La Jolla, CA, USA). One-way analysis of variance followed by Tukey’s post hoc test was applied to compare two or more groups, for example, in apoptosis assays, ESC cell cycle analysis and R-loop quantification. Data are presented as mean ± standard error of the mean. A p-value of less than 0.05 was considered statistically significant.

## Results

### INO80 deficiency sensitizes ESCs to replication stress and disrupts cell cycle progression

(A) Flow cytometry plots of Annexin V-FITC/propidium iodide (PI) staining showing apoptosis in control (CONT) and iKO ESCs, with or without hydroxyurea (HU) treatment (0.5 mM or 2 mM). (B) Quantification of apoptotic cells reveals a significant increase in apoptosis in iKO ESCs compared to that in CONT ESCs, which is further enhanced upon HU treatment (n = 4). (C) Flow cytometry histograms of PI staining showing cell cycle distribution in CONT and iKO ESCs, with or without HU treatment. (D) Quantification of cell cycle phase showing the proportion of cells in G1, S, and G2/M phases in control and iKO ESCs, with or without HU treatment (n = 4). Statistical marks represent comparisons of G1-phase ratios between groups. *p < 0.05; **p < 0.01; ***p < 0.001.

**Fig. 1:**
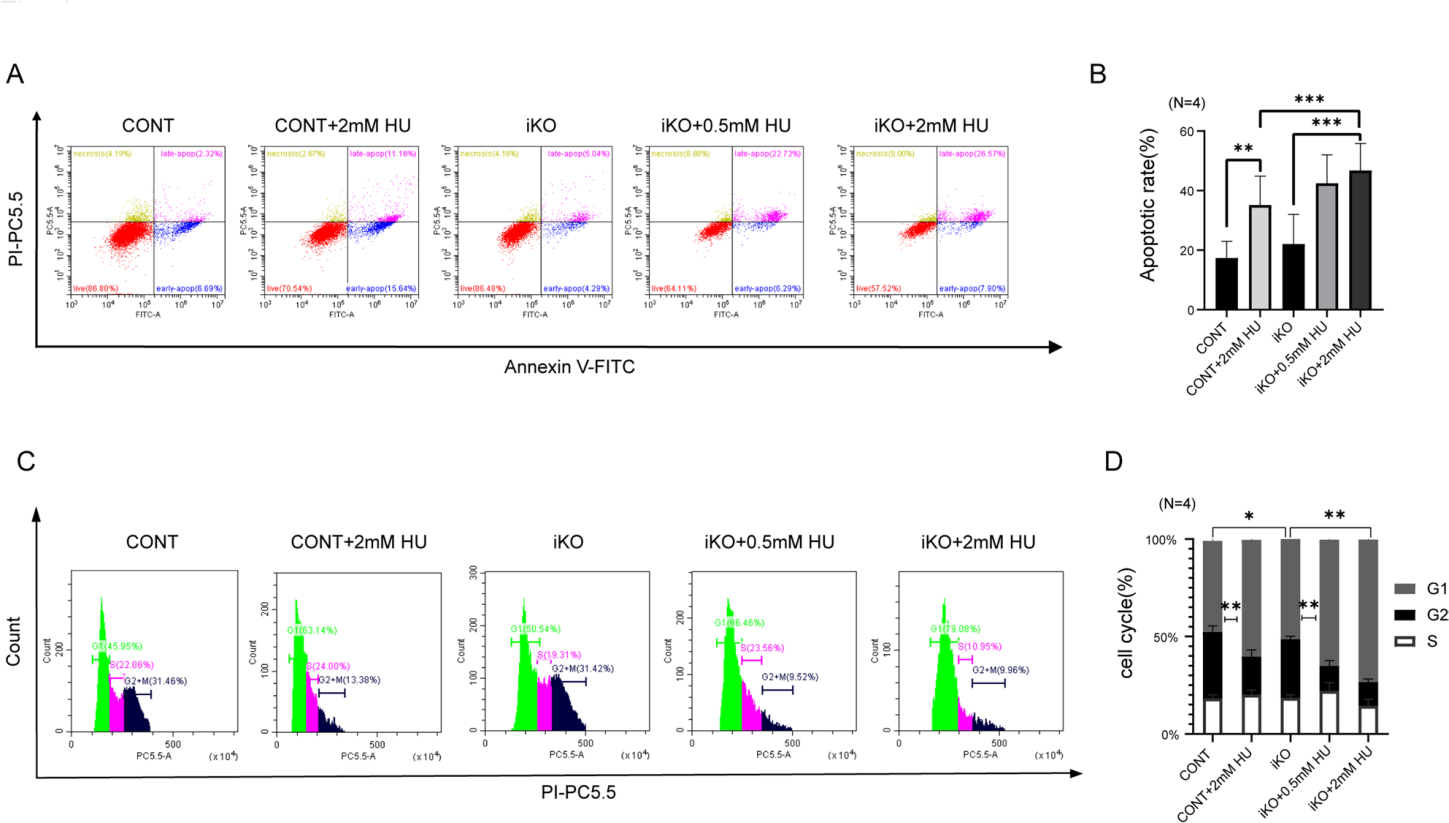
Loss of chromatin remodeler INO80 increases apoptosis and induces G1 phase cell cycle arrest in embryonic stem cells (ESCs) under replication stress

Phenotypic characterization using Annexin V/PI staining revealed a marked increase in late-stage apoptosis following INO80 deletion, with the effect being particularly pronounced under replication stress induced by hydroxyurea (HU) treatment (Fig. 1A). HU is a well-established inducer of replication stress that inhibits ribonucleotide reductase, leading to dNTP depletion and stalling of replication forks. This HU-induced replication stress system enabled us to evaluate the role of INO80 in protecting ESCs from replication-associated genomic stress. While control ESCs exhibited low basal levels of apoptosis, exposure to 2 mM HU moderately increased apoptosis. INO80 iKO ESCs exhibited a marked increase in apoptosis upon HU treatment compared to controls, indicating that the iKO cells were more vulnerable to HU-induced replication stress (Fig. 1B). These results suggest that INO80 protects ESCs against replication-associated cell death, potentially through regulation of R-loop stability and chromatin integrity.

Cell cycle profiling using PI-based flow cytometry showed that control ESCs generally displayed a typical distribution across G1, S, and G2/M phases under basal conditions. Upon HU treatment, control cells exhibited a modest increase in the G1 population, which is consistent with replication stress-induced G1 arrest (Fig. 1C, D). By comparison, INO80 iKO ESCs tended to show an increased G1 fraction and a decreased G2/M fraction even without HU, suggesting a potential impairment in the G1-to-S phase transition. This G1 accumulation appeared to be more pronounced following HU treatment, with G1 proportions exceeding 73% in the iKO + 2 mM HU group. Taken together, these observations suggest that INO80 may contribute to protecting ESCs from replication stress-associated apoptosis and may help ensure proper cell cycle progression.

### INO80 iKO causes significant loss of physiological R-loops

(A) Immunofluorescence staining of S9.6 (an R-loop marker), γ-H2AX (a DNA damage marker), OCT4 (a pluripotency marker), and DAPI (a nuclear marker) in control (CONT) and INO80 knockout (iKO) ESCs, with or without 2 mM HU treatment. Scale bar: 2 μm. (B) Quantification of S9.6-positive foci. Quantification was performed from > 50 nuclei per condition in three independent experiments. (C) Dot blot analysis of R-loops (S9.6) and double-stranded DNA (dsDNA) from CONT and iKO ESCs, with or without 2 mM HU treatment. RNase H treatment ( +) was used as a specificity control for R-loop detection. (D) Quantification of the S9.6/dsDNA signal ratio from dot blot assays.

**Fig. 2:**
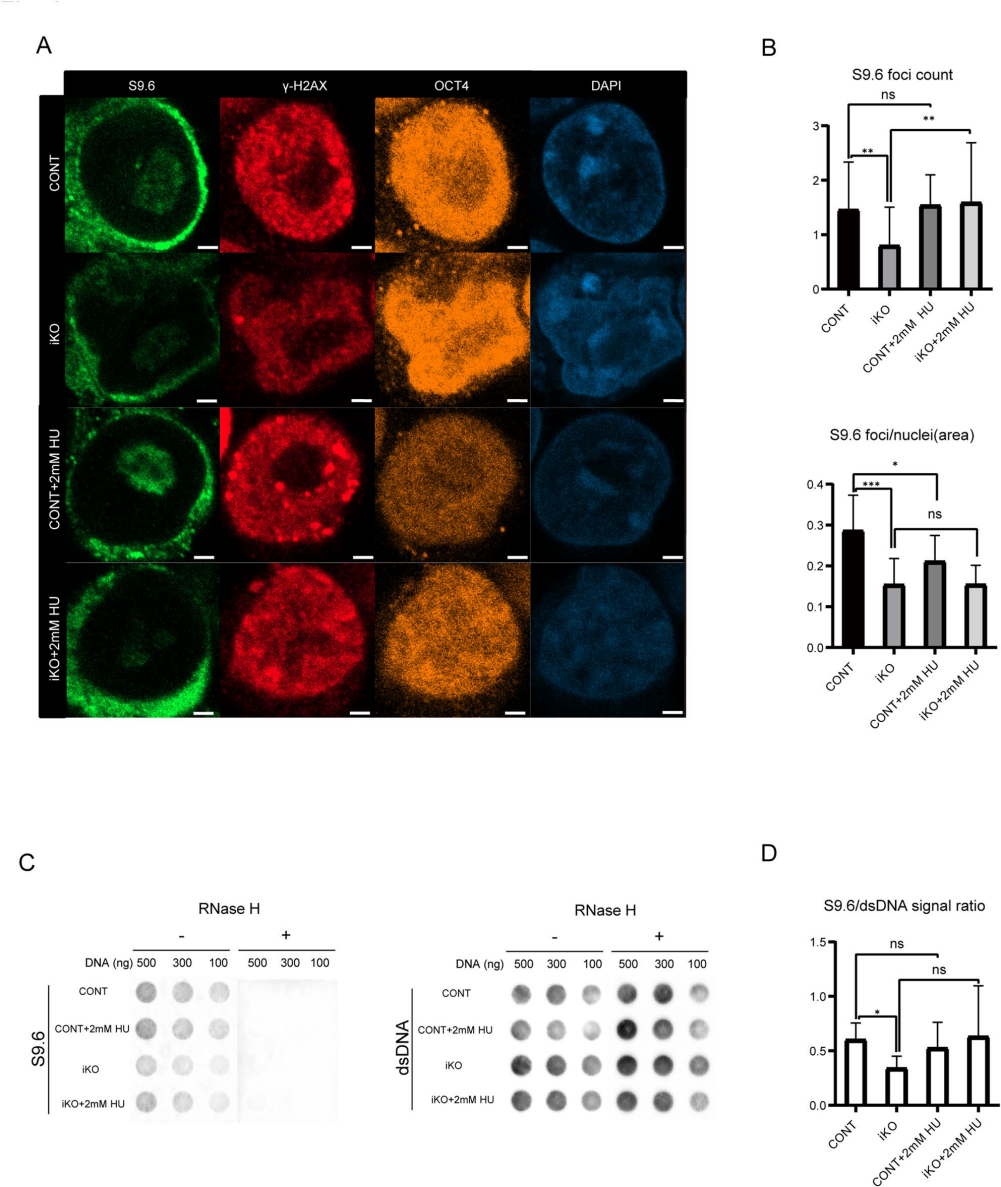
Immunofluorescence and dot blot analysis of RNA:DNA hybrid structures with displaced single-stranded DNA (also known as R-loops) in ESCs

Statistical analysis after the S9.6, γH2AX, and OCT4 immunofluorescence (Fig. 2A) revealed a significant decrease of R-loop foci numbers and foci per nucleus (Fig. 2B) in iKO over control, and a significant increase of R-loop foci numbers in iKO + HU over iKO. The immunofluorescences results were further supported by R-loop dot blot analysis (Fig. 2C). Under basal conditions, iKO ESCs displayed significantly lower S9.6 signal intensities than control cells. RNase H treatment abolished the R-loop signal, confirming RNA/DNA hybrid specificity. Quantification of the S9.6/dsDNA ratio across eight independent experiments confirmed a statistically significant reduction of R-loops in iKO ESCs (Fig. 2D). Therefore, our analysis strongly suggests that INO80 contributes to the maintenance of R-loops under physiological conditions.

Taken together, these findings indicate that INO80 contributes to the maintenance of steady-state R-loop structures formed under physiological conditions, particularly in transcriptionally active regions. In contrast, INO80 appears dispensable for R-loop accumulation induced by replication stress. This differential behavior implies that the role of INO80 in R-loop regulation is likely context-dependent, functioning more as a stabilizer than a suppressor of R-loops in ESCs.

### INO80 deletion leads to a global reduction of R-loops, particularly at promoters enriched for pluripotency-associated motifs

(A) Heatmaps and average signal profiles of R-loop CUT&Tag data (S9.6) in Control (CONT) and iKO ESCs. Signals are displayed around transcription start sites (TSS), transcription end sites, CpG islands, and ENCODE candidate cis-regulatory elements. (B) Peak counts for differential R-loop peaks, categorized into common, CONT-enriched, and iKO-enriched groups. A pie chart depicts the distribution of CONT-enriched R-loop peaks across genomic features, including promoter-TSS, 5′ UTR, exon, intron, 3′ UTR, TTS, non-coding, and intergenic regions. (C) Motif enrichment analysis of CONT-enriched R-loop peaks. The top 10 enriched motifs are shown for promoter, intron, and intergenic regions, with bubble size representing the percentage of target regions containing each motif and color indicating statistical significance. (D) Gene Ontology (GO) enrichment analysis of genes associated with R-loop loss in promoter and intronic regions. The top enriched biological processes are shown, including transcriptional regulation, chromatin remodeling, DNA repair, and developmental pathways. Circle size indicates the number of genes, and color represents fold enrichment.

Previous studies have demonstrated the role of DNA repair factors in regulating RNA:DNA hybrid homeostasis, particularly during replication fork stalling, double-strand break (DSB) formation, and post-replicative repair [39–42, 57]. These findings underscore the importance of DNA damage response (DDR) components in controlling R-loop accumulation and genomic integrity maintenance.

**Fig. 3:**
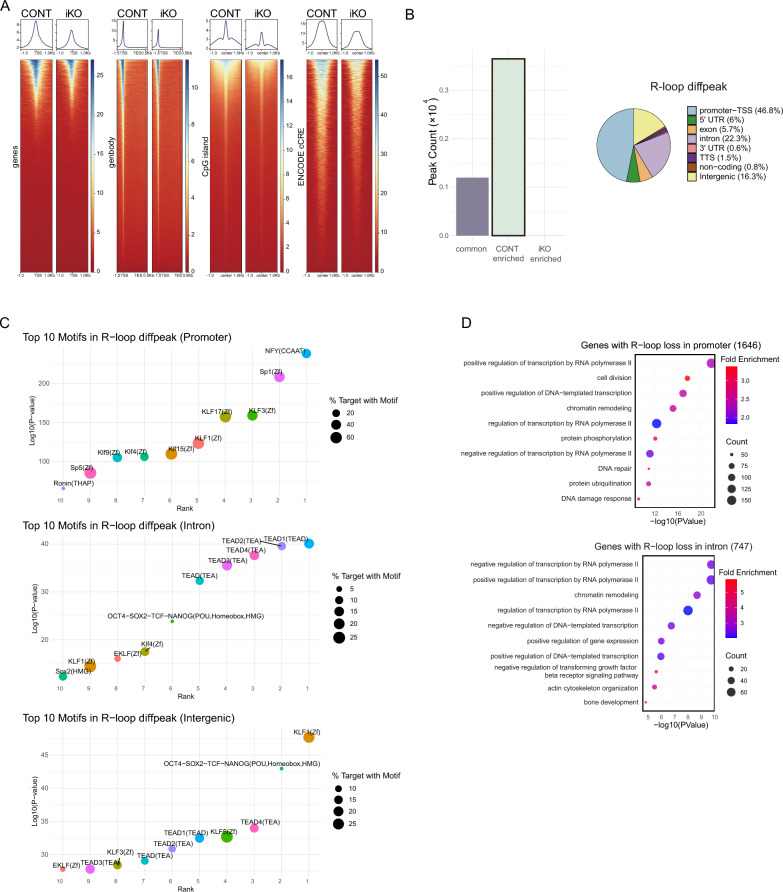
Genome-wide analysis of R-loop redistribution upon INO80 loss

To assess the genome-wide distribution of R-loops in the presence or absence of INO80, we performed CUT&Tag analysis using the S9.6 antibody. In contrast to the well-characterized R-loop accumulation observed in cancer cells following chromatin remodeler loss, ESCs exhibit a potentially different pattern (Fig. 3A). Deletion of the INO80 complex in ESCs led to a global reduction in R-loop signal intensity, as confirmed by differential peak analysis, which showed that all changes reflected signal loss rather than gain. No new R-loop-enriched regions emerged, indicating that INO80 is essential for the maintenance of pre-existing R-loop structures. This reduction was particularly prominent at promoter regions, which accounted for the largest proportion (46.8%) of affected peaks (Fig. 3B).

To further characterize the regulatory landscape of altered R-loop regions, we conducted motif enrichment analysis on differential CUT&Tag peaks grouped by their genomic features (Fig. 3C). In promoter-associated regions, motifs for canonical transcription factors such as NFY and Sp1 were highly enriched, along with several zinc finger motifs associated with the Krüppel-like factor (KLF) family, including KLF3, KLF4, and KLF17, which are known to regulate pluripotency and maintain promoter accessibility in ESCs [58]. Within intronic regions, motifs of the TEAD family (TEAD1-4), key effectors of Hippo signaling and cell fate determination, were predominant. Notably, motifs corresponding to the OCT4-SOX2-TCF-NANOG complex were detected in both intronic and intergenic regions, along with KLF1 and KLF5, indicating a strong enrichment of pluripotency-associated regulatory elements. In intergenic regions, KLF1 showed the strongest enrichment, accompanied by several TEAD motifs.

The biological relevance of regions exhibiting reduced R-loop occupancy in the absence of INO80 was assessed using GO enrichment analysis of genes associated with differential R-loop peaks (Fig. 3D). Genes linked to promoter-localized R-loop loss (n = 1646) showed strong enrichment for terms related to transcriptional regulation, including positive regulation of transcription by RNA polymerase II, chromatin remodeling, and cell division. Notably, other significantly enriched categories involved DNA repair and the DDR, suggesting that INO80-dependent R-loops at promoters contribute to genome maintenance. Similarly, genes associated with intron-localized R-loop loss (n = 747) were enriched for processes related to transcriptional regulation, particularly negative regulation of transcription and DNA-templated transcription, as well as chromatin organization and key signaling pathways. These findings indicate that INO80 contributes to R-loop homeostasis at loci functionally tied to transcription- and replication-associated genome stability. Collectively, these findings suggest that regions with reduced R-loop occupancy in INO80-deficient ESCs may be enriched for sequence motifs associated with key ESC transcriptional regulators, such as those involved in pluripotency and developmental gene control.

### Context-dependent regulation of R-loops by INO80 links chromatin remodeling to transcriptional control in ESCs

(A) Peak count distribution of differential R-loop peaks, categorized into common, CONT-enriched, and iKO-enriched groups. A pie chart illustrates the proportion of R-loop peaks with (w) or without (wo) INO80 occupancy. (B) Correlation between INO80 occupancy and R-loop distribution. (C) Motif enrichment analysis of R-loop peaks with or without INO80 binding. The top 10 enriched motifs are shown for each category, with bubble size indicating the percentage of target regions containing each motif and color representing the statistical significance (log_10_ P-value). (D) Gene Ontology (GO) enrichment analysis of genes exhibiting reduced R-loop levels. Circle size corresponds to the number of genes, and color indicates fold enrichment.

**Fig. 4:**
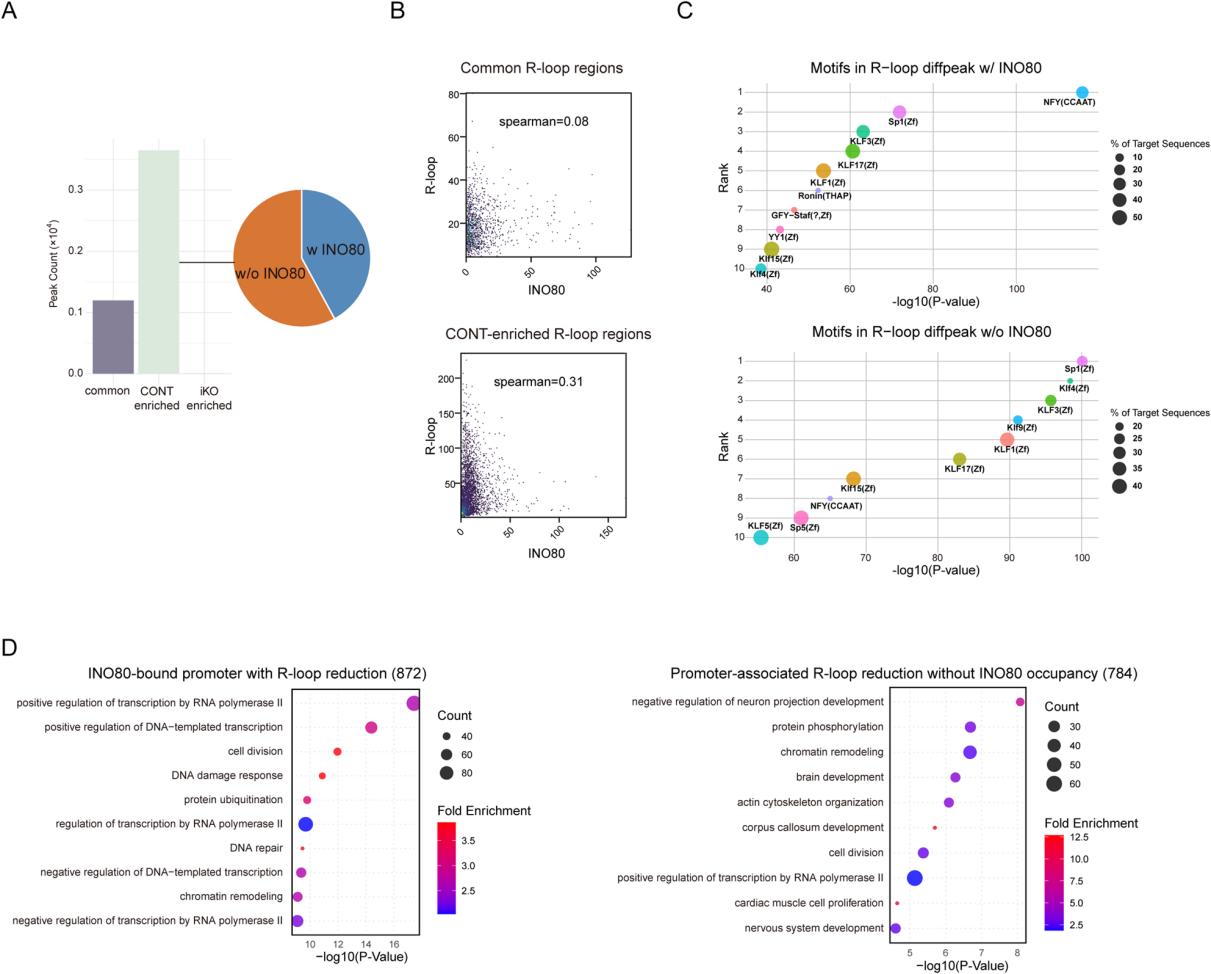
Analysis of INO80-dependent and independent regulation of R-loops

The relationship between R-loop dynamics and INO80 chromatin occupancy was examined by comparing differential R-loop peaks with INO80 ChIP-seq peaks in ESCs. Reduced R-loop signals were observed at INO80-bound loci, consistent with a possible direct role of INO80 in stabilizing R-loops at these sites (Fig. 4A and Supplementary Fig. S1A). Nonetheless, approximately two-thirds of the differential R-loop peaks occurred at regions lacking detectable INO80 occupancy. This distribution suggests that, beyond its direct binding sites, INO80 may also affect R-loop homeostasis indirectly through changes in chromatin organization or secondary transcriptional effects. Furthermore, we analyzed the correlation between INO80 occupancy and R-loop distribution in CONT-enriched and Common R-loop peaks. In CONT-enriched R-loop peaks, a weak positive correlation was observed (Spearman ρ = 0.31). On the other hand, no apparent correlation was detected within the common R-loop peaks (ρ = 0.08) (Fig. 4B).

In the subset of R-loop peaks overlapping with INO80 ChIP-seq signals (with INO80), we observed significant enrichment of motifs recognized by transcription factors such as NFY (CCAAT box-binding factor), KLF1, KLF17, Ronin (THAP family), and YY1 (Fig. 4C). Notably, NFY, the top-ranked motif in this group, is a well-known transcriptional activator that recruits histone-modifying complexes to promoters and facilitates chromatin accessibility during active transcription [59]. Similarly, KLF1 and KLF17 are members of the KLF family, which have been implicated in chromatin remodeling and stem cell fate regulation [60]. Ronin, a THAP domain-containing transcription factor, plays an established role in maintaining pluripotency gene networks [61], while YY1 is a multifunctional regulator involved in transcriptional activation. YY1 also physically interacts with chromatin remodeling complexes, including INO80, and contributes to DNA DSB repair via the homologous recombination (HR) pathway [62, 63]. Taken together, the enriched motifs in this INO80-bound group are strongly associated with transcriptional activation, chromatin architecture, and genome maintenance, consistent with the results from GO enrichment analysis.

In contrast, R-loop peaks lacking INO80 occupancy exhibited a distinct motif signature enriched for transcription factors associated with developmental regulation and differentiation-related gene expression. The most significantly enriched motif in this group was Sp1, a zinc finger transcription factor known to modulate gene expression during neural and epithelial development [64–66]. Other prominent motifs included KLF4, KLF3, KLF9, and KLF15, which are involved in stem cell fate transitions, lineage commitment, and responses to environmental signaling [67–70]. Notably, although members of the KLF family appeared in both INO80-bound and INO80-unbound groups, the specific factors differed, suggesting functional divergence. KLF1/KLF17 were enriched in the INO80-bound group and are linked to transcriptional activation and genome integrity, whereas KLF4, KLF3, and KLF9 in the INO80-unbound group are associated with repressive or lineage-specific transcriptional programs.

To investigate the functional implications of R-loop sites co-localizing with INO80, GO enrichment analysis was performed on genes associated with differential R-loop peaks that either overlapped (with INO80) or did not overlap (without INO80) with INO80 ChIP-seq signals. Among the 872 genes with R-loop peaks overlapping INO80 binding, enriched terms were primarily related to transcriptional activation and genome maintenance, including positive regulation of transcription by RNA polymerase II, DNA-templated transcription, DNA repair, and the DDR (Fig. 4D). In contrast, 784 genes linked to R-loop peaks lacking INO80 occupancy were enriched for developmental and structural processes, such as neuron projection development, brain development, and actin cytoskeleton organization, as well as chromatin remodeling and protein phosphorylation (Fig. 4D). These findings suggest that INO80 may indirectly influence R-loop homeostasis at developmental loci, potentially through changes in chromatin accessibility or secondary transcriptional effects resulting from the loss of chromatin remodeling activity.

### Transcriptomic consequences of INO80 loss at promoter-associated R-loop regions

(A) GO enrichment analysis of genes with promoter R-loop peaks overlapping INO80 binding sites. The top panel shows enriched terms for upregulated genes, and the bottom panel shows enriched terms for downregulated genes. Dot size reflects the number of genes, and color represents fold enrichment. (B) Heatmap of R-loop regulatory genes grouped by expression patterns in CONT and iKO ESCs. Hierarchical clustering of normalized expression values identifies two primary gene clusters: Cluster 1, genes upregulated in iKO ESCs and Cluster 2, genes downregulated in iKO ESCs. Heatmap showing expression profiles of genes with promoter-associated R-loops overlapping INO80 ChIP-seq peaks. The color scale indicates relative expression levels (red: high, blue: low). (C) Genome browser tracks of *Top2a* and *Ddx5* illustrate representative INO80-bound promoter regions exhibiting differential R-loop signals and transcriptional changes in response to INO80 loss.

**Fig. 5:**
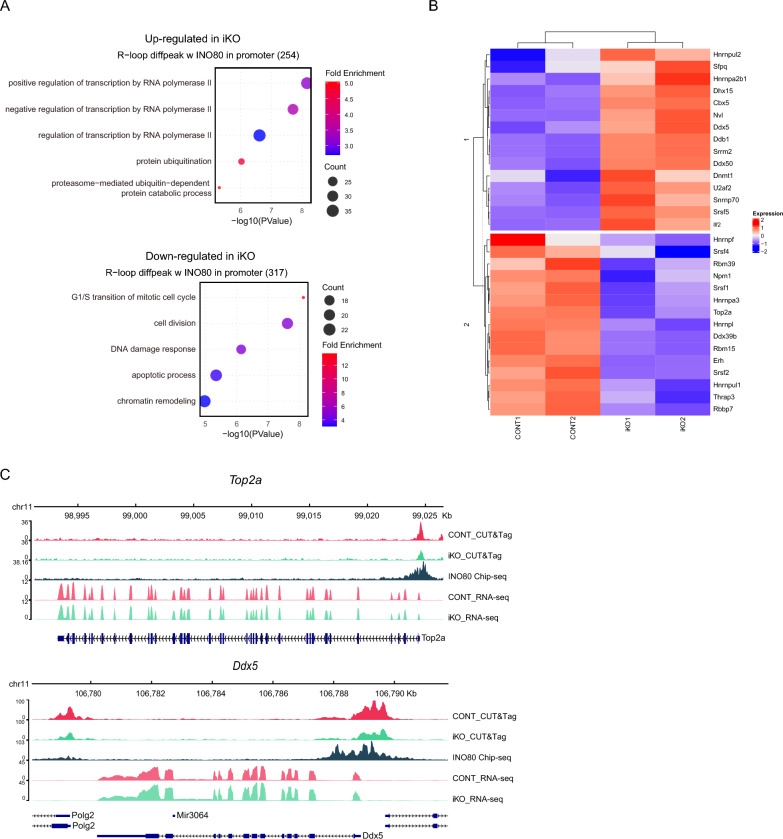
Transcriptomic consequences of INO80-associated R-loop loss

The functional impact of INO80-dependent R-loop regulation was investigated through an integrated analysis combining INO80 ChIP-seq, R-loop CUT&Tag, and RNA-seq datasets. Specifically, we focused on genes exhibiting differential R-loop peaks at promoter regions that overlapped with INO80 binding, assessing their expression profiles in INO80 knockout ESCs using publicly available RNA-seq data (GSE158545). GO enrichment analysis revealed distinct biological signatures associated with different expression patterns (Fig. 5A). Genes upregulated upon INO80 loss were enriched for processes such as regulation of transcription by RNA polymerase II, protein ubiquitination, and proteasome-mediated protein catabolism, suggesting compensatory transcriptional responses and activation of degradation pathways upon INO80 loss. In contrast, genes downregulated upon INO80 loss were associated with cell division, the G1/S transition of the mitotic cell cycle, chromatin remodeling, and the DDR. These observations collectively imply that INO80-associated R-loop regions may contribute to maintaining transcriptional programs that support ESC proliferation and genome stability, although further studies will be required to establish a direct mechanistic link. Next, transcriptomes from INO80 iKO ESCs and RNaseH1-overexpressing ESCs [71] were compared. The analysis revealed that 106 differentially expressed genes (DEGs) associated with R-loop reduction in INO80 iKO cells overlapped with the genes altered by RNaseH1 overexpression (Supplementary Fig. S2).

We mapped a curated set of R-loop-interacting protein genes [72] onto the clustered gene expression heatmap. Among the DEGs shown in Fig. 5B, multiple members of this 30-gene subset were distributed across both Cluster 1 and Cluster 2, suggesting that INO80 may modulate certain R-loop regulators at the transcriptional level. Collectively, the enrichment profiles of these gene clusters support a model in which R-loops colocalizing with INO80 contribute to transcriptional activation and cell cycle progression. Conversely, INO80 loss appears to trigger compensatory transcriptional responses and activate stress or degradation-related pathways, likely reflecting impaired maintenance of these functional R-loop sites. Genome browser tracks for *Top2a* and *Ddx5* illustrate differential R-loop signals and gene expression between control and INO80 knockout ESCs (Fig. 5C) [73]. In *Top2a*, promoter-associated R-loop signals and transcript levels were reduced in iKO ESCs, whereas in *Ddx5*, R-loop signals decreased while gene expression increased. These divergent outcomes imply that INO80-dependent R-loops can exert gene-specific regulatory effects, either reinforcing or restraining transcription depending on local promoter context. Such complexity highlights that INO80 loss not only disrupts global R-loop homeostasis but also rewires transcriptional networks in a context-dependent manner, with implications for stem cell identity and genome stability.

## Discussion

R-loops, which are three-stranded nucleic acid structures composed of an RNA:DNA hybrid and a displaced ssDNA, are increasingly recognized as regulatory elements at the intersection of transcription and genome maintenance [36]. Although historically regarded primarily as sources of genomic instability, recent studies have highlighted their physiological functions, particularly in transcriptional regulation, epigenetic programming, and chromatin organization [74]. The precise balance between R-loop formation and resolution is tightly regulated by a host of RNA-binding proteins, helicases, and chromatin remodelers, many of which act in coordination with DNA repair pathways [75]. Disruption of this balance can lead to replication stress and DNA damage, particularly in rapidly proliferating cells [76].

Among chromatin remodelers, the INO80 complex has emerged as a critical factor in maintaining genome integrity under replication stress [77]. INO80 has been shown to facilitate replication fork restart, promote HR, and resolve TRCs by modulating chromatin structure [31]. In yeast and mammalian somatic cells, INO80 loss typically results in increased R-loop accumulation and heightened genomic instability [20, 30]. However, the role of INO80 in pluripotent stem cells remains largely unexplored, despite the fact that ESCs possess uniquely open chromatin, high transcriptional activity, and rapid cell cycling-all of which may modulate R-loop dynamics differently than in somatic cells [24, 27].

The impact of INO80 loss also extended to cellular phenotypes. INO80-deficient ESCs exhibited increased late-stage apoptosis, particularly under HU-induced replication stress. These findings align with those of previous studies linking INO80 to replication fork protection and DNA repair (Fig. 1). As previously reported by our group, INO80 deficiency leads to a delayed G1/S transition in ESCs, disrupting the characteristic short G1 phase required for pluripotency maintenance [32]. In this study, we further demonstrated that this G1 arrest phenotype is evident even under unstressed conditions and is significantly exacerbated upon HU-induced replication stress. Collectively, these findings suggest that beyond its role in basal cell cycle regulation, INO80 is also required to buffer ESCs against replication stress-induced cell cycle perturbations.

We identified a striking divergence from previously described roles of INO80. In contrast to the canonical model, in which loss of the chromatin remodeler increases R-loop levels [20, 30], R-loop quantification via immunofluorescence and dot blot assays revealed a marked reduction in steady-state R-loops under basal conditions in INO80 iKO ESCs. Notably, these R-loops were partially restored upon HU treatment, even in the absence of INO80, suggesting that INO80 is dispensable for R-loop accumulation under replication stress but may be important for maintaining R-loop homeostasis under physiological conditions (Fig. 2). Previous studies reported an increase in R-loop accumulation upon HU treatment in U2OS and HeLa cells [78, 79]. On the other hand, it was shown that mESCs prevent R-loop formation by Lnc530-mediated recruitment of DDX5 and TDP-43 to resolve R-loops after HU treatment [80]. Therefore, it is likely that changes in R-loops induced by HU vary depending on cell type.

Through the integration of CUT&Tag and ChIP-seq datasets, we observed that a considerable fraction of R-loop peaks diminished upon INO80 deletion overlapped with INO80-binding sites in wild-type ESCs (Fig. 4), suggesting a direct chromatin-level contribution of INO80 to R-loop stabilization. Indeed, we observed a positive correlation between INO80 occupancy and R-loop distribution specifically at these CONT-enriched R-loop peaks, whereas no such correlation was found at common R-loop peaks (Fig. 4).

However, a substantial proportion of R-loop loss occurred at loci lacking INO80 occupancy, implying indirect regulation through global chromatin reorganization or secondary transcriptional changes following INO80 disruption. GO enrichment analysis of these co-occupied regions revealed significant associations with transcriptional activation, epigenetic regulation, and DNA damage repair, supporting a potential role for INO80 in preserving the integrity of regulatory genomic regions. In contrast, loci exhibiting R-loop loss in the absence of detectable INO80 binding were enriched for developmental and cytoskeletal genes, suggesting that INO80 may also indirectly influence R-loops through large-scale chromatin reorganization or secondary transcriptional effects (Fig. 4). Collectively, these observations point to both locus-specific and genome-wide contributions of INO80 to R-loop maintenance rather than direct regulation alone.

Transcriptome profiling identified two major gene clusters exhibiting distinct transcriptional responses to INO80 loss. Genes upregulated upon INO80 loss were enriched for pathways related to transcriptional regulation, possibly reflecting a compensatory reorganization of gene expression programs associated with R-loop destabilization. In contrast, genes downregulated following INO80 loss were associated with pathways involved in cell division, G1/S cell cycle transition, chromatin remodeling, and the DDR. These findings suggest that INO80-associated R-loops may contribute to sustaining transcriptional networks that support ESC proliferation and genomic stability. Notably, only 106 genes overlapped between genes showing decreased R-loop levels in an INO80-dependent manner and genes responsive to RNaseH1 overexpression [71] (Supplementary Fig. S2). The result suggests that RNaseH1 functions as a global R-loop resolver, whereas INO80 modulates R-loop dynamics at specific subsets of genomic regions. The non-overlapping genes may reflect general roles for INO80 in the regulation of chromatin remodeling and DNA repair.

Ninety-six of 205 R-loop-interacting proteins [72] were identified as direct interactors of the INO80 complex [81], supporting a potential role for INO80 in recruiting the factors to target genomic regions. In addition, to assess whether INO80 modulates established R-loop regulatory factors, we cross-referenced the DEGs with a curated set of R-loop interacting proteins previously identified through proteomic analyses. Among these, 30 genes overlapped with INO80-associated promoter R-loop differential peaks (Fig. 5). Representative genome browser tracks further supported this interpretation. For instance, *Top2a*, a gene involved in resolving transcription-induced topological stress, exhibited decreased R-loop levels and transcript abundance upon INO80 loss. In contrast, *Ddx5*, a known R-loop-resolving helicase, showed reduced R-loop signals but increased transcription. These divergent patterns underscore gene-specific transcriptional responses to R-loop disruption, which may reflect differences in promoter architecture, chromatin accessibility, or transcription factor recruitment dynamics between these loci.

Under replication stress induced by HU treatment, R-loop levels were restored in INO80 iKO ESCs, indicating that stress-induced R-loop formation can occur through alternative mechanisms independent of INO80. Previous studies have shown that Mec1/ATR cooperates with INO80 and the transcription elongation complex PAF1C to resolve TRCs by modulating RNA polymerase II occupancy under stress conditions [45, 82]. Mec1/ATR and PAF1C may function as compensatory pathways to facilitate replication fork progression in the absence of INO80, thereby forming part of a broader genome surveillance network that coordinates R-loop regulation with replication stress responses.

Notably, HU treatment restored R-loop levels to near-control values in iKO ESCs, indicating that INO80 is dispensable for replication stress-induced R-loop formation but may be required for the stabilization of functional, co-transcriptional R-loops under normal conditions. Although R-loops have traditionally been regarded as transcriptional byproducts that threaten genome stability, recent studies have suggested a more nuanced relationship between R-loop formation and the DDR. Wahba et al. [83] demonstrated that Rad51-mediated strand invasion can generate R-loops at homologous loci independent of local transcription. Consistently, removal of RNA:DNA hybrids through RNase H overexpression impairs HR in yeast [84] and disrupts both HR and non-homologous end joining in human cells [85]. Notably, a recent study by Keil et al. [15] showed that INO80 plays a critical role in facilitating HR by remodeling chromatin at DSB sites, particularly during symmetric divisions of neural progenitors. Collectively, these findings suggest that INO80 not only regulates R-loops at transcriptionally active loci but also modulates trans-acting R-loops through its involvement in HR-dependent DNA repair pathways [86–88].

Given the observed genome-wide reduction in R-loop levels upon INO80 loss in ESCs, INO80 may contribute to stabilizing specific cis-formed R-loops and may also influence a subset of trans-acting R-loops that arise during replication, transcription, or HR processes [83, 89, 90]. This context-dependent R-loop stabilization could be particularly important in ESCs, which are characterized by high proliferative capacity, elevated recombination activity, and a globally open chromatin state-features that predispose them to TRCs and HR-dependent genome maintenance [91, 92]. These findings support a model in which INO80 stabilizes functional R-loops and preserves both transcriptional programs and genomic integrity under both normal and stress conditions.

## Conclusions

This study identifies a previously unrecognized role for the INO80 chromatin remodeling complex in maintaining physiological R-loop homeostasis in ESCs. INO80 stabilizes steady-state R-loops, particularly at promoters enriched in pluripotency-associated transcription factors, rather than acting solely as a resolver of harmful RNA:DNA hybrids. Loss of INO80 leads to a global reduction in R-loop levels, heightened sensitivity to replication stress, G1 phase arrest, and altered transcriptional programs essential for genome integrity. Overall, our findings highlight INO80 as a key regulator of R-loop-mediated transcriptional and chromatin dynamics, offering new insights into genome maintenance mechanisms in pluripotent cells.

## Supplementary Information

Below is the link to the electronic supplementary material.

Supplementary Material 1.

## Data Availability

Raw data and processed data are available from NCBI GEO accession GSE306053. All data supporting the findings of this study are included in this article and supplementary information files.

## Abbreviations

ESCs: Embryonic stem cells
CDK: Cyclin-dependent kinase
CKI: Cyclin-dependent kinase inhibitor
TSS: Transcription start site
ssDNA: Single-stranded DNA
dsDNA: Double-stranded DNA
HU: Hydroxyurea
PI: Propidium iodide
DSB: Double-strand break
HR: Homologous recombination
TRC: Transcription-replication conflict
DDR: DNA damage response
CUT&Tag: Cleavage under targets and tagmentation
ChIP-seq: Chromatin immunoprecipitation followed by sequencing
RNA-seq: RNA sequencing
CPM: Counts per million
GO: Gene ontology
HOMER: Hypergeometric optimization of motif enrichment
DEGs: Differentially expressed genes
PBS: Phosphate-buffered saline
RT: Room temperature
