# Correction: INO80 regulates promoter-associated R-loops to coordinate transcription and maintain genome stability in embryonic stem cells

**DOI:** 10.1186/s40659-026-00701-1

**Published:** 2026-05-28

**Authors:** Hoseong Lim, Ohbeom Kwon, Hyeonwoo La, Hyeonji Lee, Heeji Lee, Jeong-Tae Do, Hyuk Song, Youngsok Choi, Kwonho Hong

**Affiliations:** Department of Stem Cell and Regenerative Biotechnology and Institute of Advanced Regenerative Science, Konkuk University, Seoul, 05029 Korea

**Correction to: Biological Research (2026) 59:7** 10.1186/s40659-025-00666-7

In the sentence beginning ' Genomic DNA was extracted…' in this article, the text ' Approximately 5 μg of purified DNA was resuspended in nuclease-free water to a final volume of 50 μL and applied onto a positively charged nylon membrane (Hybond N+, RPN303B; Cytiva, Marlborough, MA, USA) using a MiniFold-1 dot blot apparatus (Cytiva). ' should have read ' Approximately 5 μg of purified DNA was divided into two tubes. One was treated with RNase H (10 U per µg DNA; M0297L, NEB, Ipswich, MA, USA), whereas the other was remained untreated as a control. Then, the RNase H-treated and -untreated samples were serially diluted and loaded onto a positively charged nylon membrane (Hybond N+, RPN303B; Cytiva, Marlborough, MA, USA) at 500, 300, and 100 ng per dot, using a MiniFold-1 dot blot apparatus (Cytiva)’.

The original article has been corrected.

