# Supplementary material for: INO80 regulates promoter-associated R-loops to coordinate transcription and maintain genome stability in embryonic stem cells
Source: Biol Res. 2026 Jan 3;59:7. doi: 10.1186/s40659-025-00666-7 (PMC12865974; doi:10.1186/s40659-025-00666-7)
Supplement: Supplementary file 1 — Supplementary Material 1. [file 40659_2025_666_MOESM1_ESM.docx]

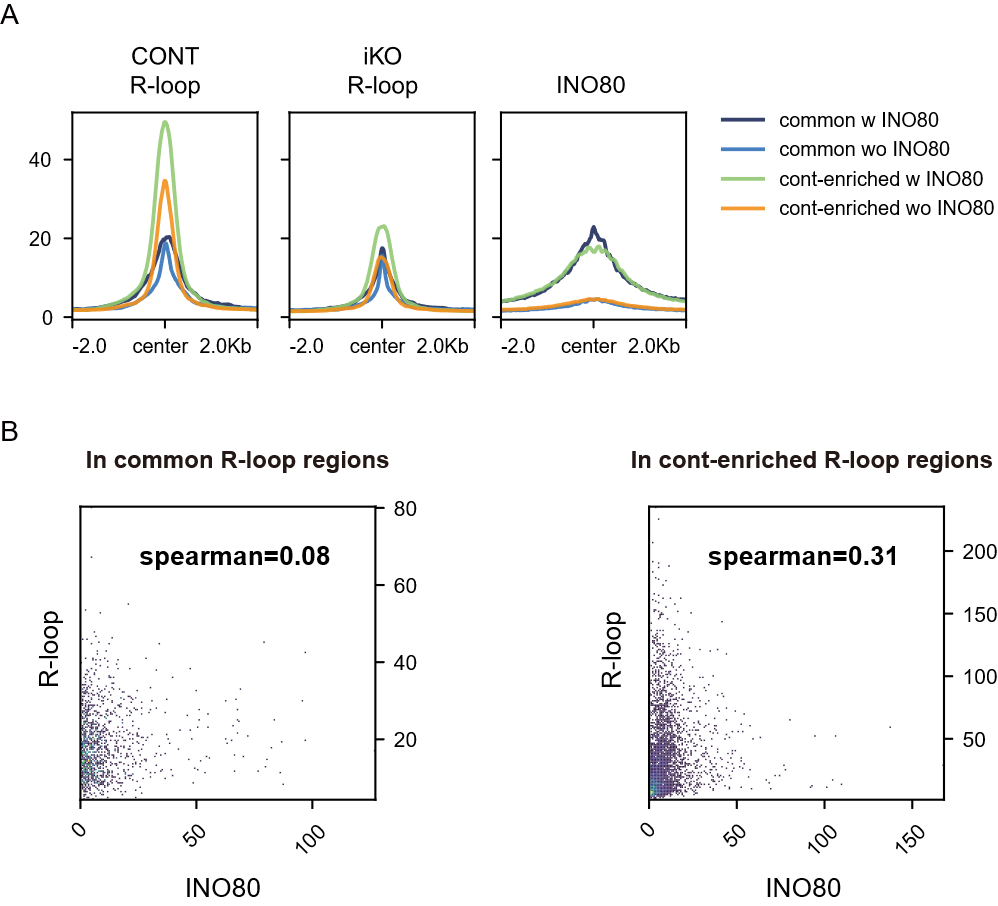


Supplementary figure.1 INO80 occupancy associates with elevated R-loop signal at CONT-enriched-but not common-R-loop sites in ESCs

1. Comparison of R-loop and INO80 signals at each locus (Common R-loop peak with INO80, Common R-loop peak without INO80, CONT-enriched R-loop peak with INO80, CONT-enriched R-loop peak without INO80).


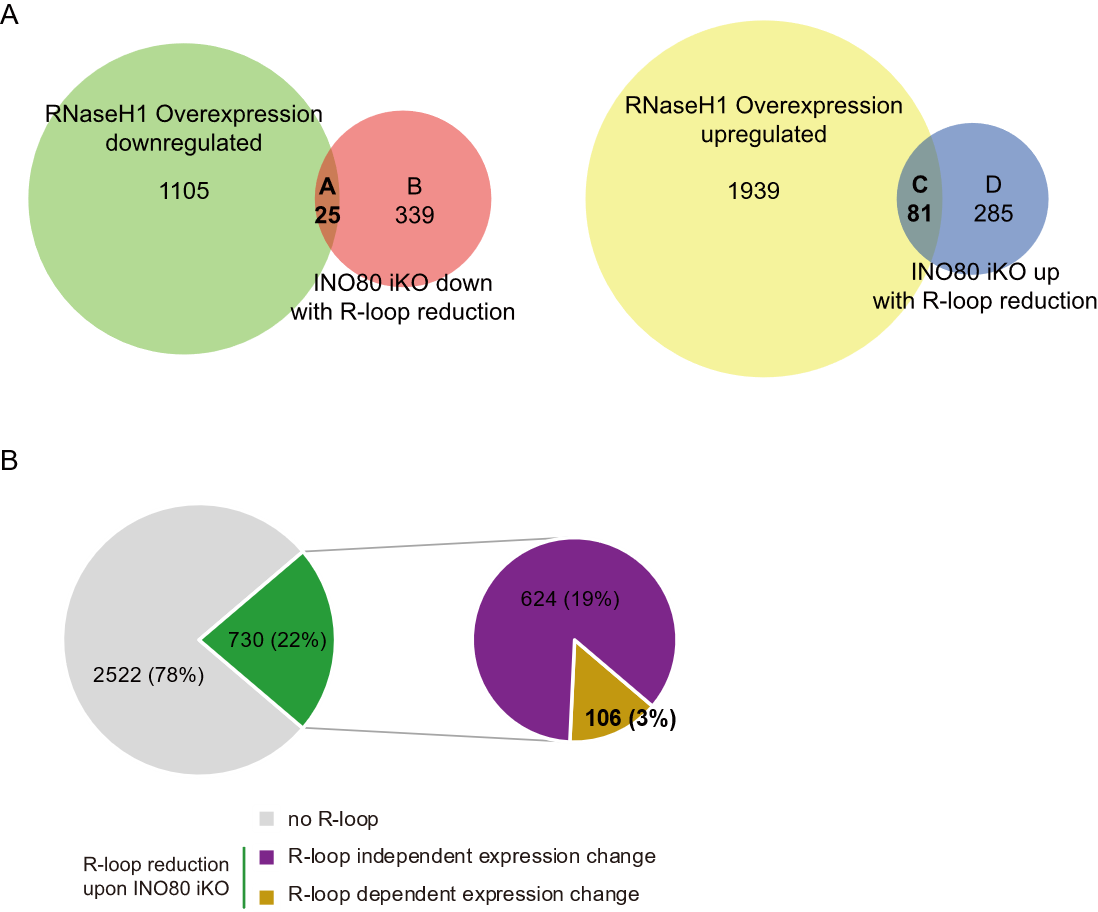


Supplementary figure 2. Identification of R-loop-dependent gene expression changes through comparison of INO80 iKO and RNaseH1 overexpression.

1. Venn diagrams comparing INO80 iKO DEGs with reduced R-loop levels and DEGs in RNaseH1-overexpressing ESCs (GSE67584).
2. Pie charts quantifying the proportion of R-loop-dependent DEGs.
